# Predictors of postpartum contraceptive use in rural Tigray region, northern Ethiopia: a multilevel analysis

**DOI:** 10.1186/s12889-018-5941-4

**Published:** 2018-08-16

**Authors:** Teklehaymanot Huluf Abraha, Berhe Beyene Gebrezgiabher, Berihu Gidey Aregawi, Desta Siyoum Belay, Lidiya Tsegay Tikue, Getachew Mebrahtu Welay

**Affiliations:** 1 Department of Reproductive Health, School of Public Health, College of Health Sciences, Aksum University, P.O. Box: 1010, Aksum, Tigray Ethiopia; 2 Department of Epidemiology & Biostatistics, School of Public Health, College of Health Sciences, Aksum University, Aksum, Tigray Ethiopia; 3 Department of Human Nutrition, School of Public Health, College of Health Sciences, Aksum University, Aksum, Tigray Ethiopia; 4 School of Nursing, College of Health Sciences, Mekelle University, Mekelle, Tigray Ethiopia; 5 Department of Nursing, Defence Force University, College of Health Science, Addis Ababa, Ethiopia

**Keywords:** Predictors, Contraception, Postpartum period, Multilevel analysis, Tigray, Ethiopia

## Abstract

**Background:**

Postpartum family planning services is one of the recommended public health intervention aimed at reducing maternal and child morbidity and mortalities. However, there is a paucity studies in rural Tigray region. Therefore, determining the level and associated factors of contraceptive use among postpartum women has the potential to contribute in achieving the Ethiopian Health Sector Transformation Plan and to the Sustainable Development Goals on maternal and infant survival.

**Methods:**

A community-based cross-sectional study was done among 1109 postpartum women from March 29, 2017 to April 29, 2017. Face –to–face interview was used for data collection. The collected data were entered and cleaned using EPI - INFO version 7statistical software and later exported to and analyzed using STATA version 12. Mixed-effects multilevel logistic regression analysis was used to identify the individual and community-level factors associated with contraception adoption. A two side *p*-value< 0.05 was considered to be statistically significant.

**Results:**

The level of contraceptive use was 38.3%. Individual-level variables such as women belong to fourth (AOR = 1.2; 95% CI: 1.1–3.2) and fifth (AOR = 1.5; 95% CI: 1.3–2.5) wealth quintiles were identified as key predictors of contraception use. In addition, partner secondary (AOR = 2.3; 95% CI: 1.8–3.5) and diploma (AOR = 1.2; 95% CI, 1.1–2.6) educational-level and postnatal care (AOR = 2.0; 95% CI: 1.9, 4.3) were also significantly affected contraception use. Community-level variables such as high community-level antenatal care services use (AOR = 2.1; 95% CI: 1.9–4.2) and proximity of women to health facility (AOR = 3.0; 95% CI: 2.7–4.6) were also determinants of contraception uptake.

**Conclusions:**

The status of contraceptive use in rural Tigray region was found to be low. It was found that both individual and community-level variables showed a marked determinant on postpartum contraception use. This study suggested that in order to increase contraceptive use the government should focus on increasing postnatal care, antenatal care services use and reduction of poverty level are important avenues for intervention.

## Background

Maternal health issue is remains a global challenge [1, 2]; complication related with pregnancy and childbirth is the leading cause of mortality and morbidity among women in their reproductive age period [3]. Every day, around 830 women die from preventable cause’s related pregnancy and childbirth. Of maternal mortality, almost all (99%) happened in developing countries [3]. In Ethiopia, maternal mortality is an important public health issue; 412 per 100,000 live births are dying [4], among the highest in the Sub-Saharan Africa (SSA) countries.

Postpartum family planning (PPFP) mainly focuses on the prevention of unintended and closed spaced pregnancies through the first one year following childbirth [5, 6]. An increase in contraception adoption during the postpartum period substantially reduces the rate of maternal and infant mortality [7]. A study done among women within one year of their last birth found that 95% of the women in middle and low income countries want to delay pregnancy within the next two years, but 70% are not yet using contraception [8].

In developing countries, if all unmet need for contraception are satisfied three-quarters would be declined in unintended pregnancies [9]. Existing studies show that if couples space their pregnancies more than 2 years apart, contraception can avert more than 30% of maternal mortalities and 10% of child deaths [10, 11]. However, the largest proportion of women with an unmet need for contraception is found among those in their first year after childbirth [8].

Accordingly, the World Health Organization (WHO) recommended that the interval between and attempt to the next pregnancy should be 24 months [12] since short birth intervals are associated with adverse pregnancy outcomes: induced abortions, miscarriage, preterm births, neonatal and child mortalities, still births and maternal depletion syndrome [13].

In Ethiopia, the higher proportions of unplanned pregnancies are due to short birth intervals [14] and low contraceptive utilization [4]. Based on the research findings approximately 21% of births happened due to short intervals of less than 24 months, and other 35% occur between 24 and 35 months [14]. In this context, postpartum period is important particularly for initiating contraception to space births in a healthy manner [5, 6, 15]. The Federal Democratic Republic of Ethiopia (FDRE) expanded and promoting through community-based distribution of family planning services at the women’s door level through health extension program since 2002 [16]. The government has removed all duty and taxes on the imported contraceptive and are available free of charge at public health care facilities since family planning is considered as key for enhancing attainment of the nation’s development goals [17]. Despite these interventions, the unmet need for contraceptive among postpartum women remains high (86%) [18]. There are a number of safe and effective family planning methods that can begin at various points after giving birth, including those used immediately postpartum to optimize birth spacing [19]. Addressing contraception issue in the postpartum period has a positive effect on maternal, neonatal, infant and child health survival because it enables women to achieve the ideal birth spacing [13, 20].

In Ethiopia, research finding are concentrated only on the micro-level characteristics [21–23] and have been positively associated with postpartum contraceptive use (e.g., secondary and above level of the mother’s educational, delivered with the assistance of a skilled attendant, attended postnatal care services). But associations with macro-level characteristics (e.g., district-level of antenatal care use, distance, sources of income and district residence) have largely been under looked. Furthermore, most of the studies on postpartum contraception use have applied single-level analytic techniques. Analyzing variables at the macro-level as if they were micro-level variables using the standard binary logistic regression model leads to loss of power. The traditional approach also suffers from a problem of analysis at the inappropriate level (atomistic or ecological fallacy) [24, 25].

The utilization of contraception is a complex scenario and influenced by different predictors [26, 27]. Individual characteristics interact with community variables to which individuals belong, meaning that women are influenced by their social contexts and that the characteristics of those communities are in turn affected by the individual variables that make up that community. However, most research evidence [21–23] did not addressed how predictors influence across the levels by applying a multilevel logistic regression analysis.

Therefore, the purpose of this study was to fill these gaps by assessing the use level and to estimate the effects (fixed and random) of individual and community-level factors with postpartum contraception use in the Tigray region. The finding from this research could help planners, programmers and decision makers to have a good insight of determinants of contraceptive use and to take appropriate intervention and this may increasing in the use of contraception methods, and a reduction of unintended pregnancies, maternal and child mortality rates.

## Methods

### Study design and setup

A community-based cross-sectional study was conducted from March 29, 2017 to April 29, 2017 in Central Zone of Tigray region. Four districts were selected from the 12 districts by lottery method. According to the 2017 Tigray Regional Health Bureau (TRHB) annual report, there were 1 comprehensive and specialized referral hospital, 1 infant comprehensive and specialized referral hospital, 17 general hospitals, 224 health centers and 668 health posts. Each cluster (*Kebele*) has a health post to provide maternal, child and reproductive health services. The Tigray regional state has made a tremendous achievements in improving maternal and child health services such as: currently unmet need for family planning in general population dropped to 18%, percentage with 4^+^ antenatal care visits (56.5%), institutional delivery (56.9%), postnatal care services utilization (45.4%), under-5 child mortality 59 per 1000 live births and magnitude of women age 15–49 have ever had an obstetric fistula 1.1% [4]. The Maternal Mortality Ratio (MMR) is 266 deaths per 100,000 live births [28].

### Sample size determination

A single population proportion formula was applied to calculate the sample size required in the study. The following assumptions were considered to determine sample size required for the study. As there is no study done in rural Tigary region in particular and Ethiopia in general, the prevalence of women using contraceptives in the extended postpartum period was assumed to be 50%, a 95% Confidence Interval (CI), a 5% margin of error (d) [29] and a design effect (DE) of 3. The sample size was also increased by 5% for none-response rate. Accordingly, the estimated sample size was 1109.

### Study population and sampling techniques

All postpartum women who gave birth in the last 12 months prior to the study period in randomly selected clusters were taken as study population. A multi-stage cluster sampling technique was applied to select the study participants. Initially, four districts of the Central Zone of Tigray, namely *Rural Adwa, Tanquae-Abrgelle, Tahtay-Maychew and Laelay-Maychew* districts/*woredas* were selected randomly. A total of twelve clusters (three clusters per district) were selected randomly. All eligible women who gave birth within the past one year preceding the survey who were found in the selected clusters were studied using house-to-house survey.

### Instruments

The main outcome variable for this study was postpartum contraceptive use and the independent variables were categorized in to two levels: Level one (lower- level variables) included the individual characteristics such as: socio-demographic, wealth index, maternal health use, infant sex and birth order. Level two (higher- level variables) included community variables such as distance to health facility, district residence, source of income and district- level of antenatal care use. The detail measurement variables are indicated in Table 1.

**Table 1:** Description and measurement of variables included in the mixed effect multilevel model, rural Tigray region, Ethiopia 2017

Variables	Description and measurements
Outcome variable	
Postpartum contraceptive use	This is defined as a women’s use of any modern method of contraception during the 12 months following their most recent childbirth. The outcome variable categorized as yes response is coded’1′ and no as ‘0′.
Individual-level variables	Level 1- determinant variables
Marital status	Marital status of respondent [currently married and un married].
Maternal age	Self reported age of respondent at the time of the survey [15–49 years].
Maternal and husband educational-level	Highest level of education level [None/Primary; Secondary or higher].
Birth order	Birth order of women’s most recent birth [index birth]. It is divided in to three categories: first and second birth, 3rd– 4th and ≥ 5th.
Birth interval	Birth interval is the length of time between two successive live births. It is divided in to three: < 24 months, 24–36 months and > 36 months.
Parity	Number of children ever given birth [None; One or more].
Wealth index	This is computed by principal component analyses from ten variables (based on the ownership of a farmland, own toilet facility, bank account, mobile phone, electricity, corrugated iron sheet roof, number of cows/oxen, horses/mules/donkeys, goats/sheep and chicken). The wealth index is categorized in to five groups and ranked from poorest (first) to wealthiest (fifth) quintile.
ANC use	Proportion of women who have received ANC at least one and above visit either at health center, hospital or health post.
Place of delivery	Proportion of women who have given birth at either at health center, hospital or health post.
PNC use	Proportion of women who have postnatal care at least one visit either at health center, hospital or health post.
Received FP counseling during prenatal	Any study participants who received family planning (FP) counseling during prenatal, yes ‘responses are coded as ‘1′ and no response as ‘0′.
History of complication during delivery	An individual study participants who have severe pre-eclampsia, eclampsia, bleeding during intra partum, abnormal presentation of the fetus, prolonged labour, obstructed labour, breech delivery, and cervical tear, if the study participants experienced at least any one of the above, it is categorized as 1(yes), if not 0 (no).
Model household	A household that applied all health extension packages at their home and got certificates of appreciations from health extension workers. Yes ‘responses are coded as ‘1′ and no response as ‘0′.
Community-level variables	Level 2-determinant variables
District -level of ANC use	Proportion of women in the district received 4^+^ANC and above visit. It is categorized in to two: high and low-level.
Source of income	The cluster’s population main source of income. It is categorized in to two: farming and mixed (farming & trading).
Distance	Distance in kilometer of villages in each cluster to the nearest health facility. It is categorized in-to three: < 1 km, 1–5 km and > 5 km.
District residence	Current district residence [*Rural Adwa, Tanquae-Abrgelle, Laelay -Maychew and Tahtay- Maychew]*.

### Data collection process

A structured and pre-tested questionnaire was used to collect the quantitative data. It was prepared first in English and then translated into ‘*Tigrigna’*, the local language. The tools were adapted from related kinds of studies [23, 26, 30]. Trained twelve BSc Nurse holders conducted the face- to- face interview and four MSc holders with a health background were supervised the data collection process. A field supervisor reviewed the questionnaires for completeness, quality and consistency at the end of each day while the investigators made periodic checks to ensure the quality of data collection process.

### Data analysis and modeling

The collected data were entered and cleaned using EPI - INFO version 7 statistical software [31] and later exported to and analyzed using STATA version 12 [32]. Descriptive statistics was performed to determine the frequency of the outcome variable and the explanatory variables. The frequency and percentage of each category within the predictor variables was obtained. This study employed mixed-effects multilevel logistic regression analysis to identify the individual and community-level factors associated with contraception use. Multilevel model analysis considered into account the hierarchical structure of the data and clustering of responses at the different levels [25]. During the statistical analysis, the characteristics of postpartum women was considered as individual-level (lower-level variables) and characteristics of the clusters were taken as level two (higher- level variables).

In this study, the following equation elaborates the multilevel analysis for postpartum contraceptive use:1

The two-level model was performed to assess the predictors of individual and community-level variables for contraception use. Where i and j are the level 1 (individual) and level 2 (community) units respectively; P_ij_ is the probability of the outcome of interest for woman i in the cluster j; the b’s are the fixed coefficients (AOR); I and C refer to individual and community-level independent variables, respectively; and u indicate the random effects for the j^th^ cluster. The error term, ε, shows unmeasured factors that may influence the primary outcome interest. In the model 1, no exposure variables were included in order to indicate the total variance in the uptake of postpartum contraception between community clusters. In the model 4, fixed effects at individual-level were included together with the community-level random effects. Finally, the results of fixed effects were presented as adjusted odds ratio (AOR) along with 95% confidence intervals (CI) and random effects as variance coefficients (VPC). The independent variables were retained in each of the models if the *p*- value< 0.2. A two side *p*-value < 0.05 was considered to be statistically significant. The variance coefficients were assessed as shown:

2 $$ VPC=\frac{\upsigma 2\ \mathrm{uo}}{\upsigma 2\ \mathrm{uo}+\frac{\uppi 2}{3}} $$

Where, π^2^/3 denotes the variance between mother from the same district (individual-level) and σ^2^u_0_ is the variance between districts (community-level variance). It gives how much of the variance is explained at the community-level [33].

## Results

### Socio-demographic characteristics

Table 2 summarizes the socio-demographic characteristics of the study participants. A total of 1109 postpartum women were interviewed. All of the study participants were between 16 and 49 years old. The women’s mean (±SD) age was 28.7(±6.4) years. All of the samples were *Tigriyan* by ethnicity. Forty-two percent of the mothers did not have any formal education while 42.7% of them attended primary-level education and 15.7% of them completed secondary or higher-level education. The majority of the interviewed mothers (80%) and their partners (86.1%) were farmers by occupation.

**Table 2:** Socio-demographic characteristics in rural Tigray region, northern Ethiopia, August 2017(*n* = 1109)

Variables	Frequency (*n*)	Percentage (%)
Maternal age (years)		
16–19	52	4.7
20–24	278	25.1
25–29	302	27.2
30–34	228	20.6
35–39	182	16.4
> =40	67	6.0
Maternal education		
Not educated	462	41.7
Primary school	473	42.7
Secondary school	162	14.6
Diploma and above	12	1.1
Partner/husband education		
Not educated	364	32.8
Primary school	541	48.8
Secondary school	176	15.9
Diploma and above	28	2.5
Marital status		
Currently married	1047	94.4
Not currently married	62	5.6
Maternal occupation		
House wife	126	11.4
Farmer	900	81.2
Others^a^	83	7.5
Husband/partner occupation		
Farmer	955	86.1
Private worker	62	5.6
Others^a^	92	8.3
Wealth index		
Lowest	220	19.8
Second	227	20.5
Middle	213	19.2
Fourth	225	20.3
Highest	224	20.2
Infant sex		
Male	470	42.4
Female	639	57.6
Birth order		
1–2	417	47.6
3–4	427	38.5
5^a^	265	23.9

^a^(Daily labor, private worker, student, teacher)

### Characteristics of maternal health services use

One thousand eighteen (91.79%) attended at least one antenatal visit and 547(49.3%; 95% CI: 46.3–52.3%) attended the World Health Organization (WHO) recommended four visits. Nine hundred forty five (85.2%) of the mothers were reported that they delivered at health institutions. Seven hundred and two hundred forty five of the study participants gave birth at health centers and hospitals respectively. Out of the total interviewed women: one hundred fifty seven (14.0%; 95% CI: 12.3–16.3%), one hundred ninety three (17.4%; 95% CI: 15.1–19.6%) and one hundred fifty (13.5%; 95% CI: 11.5–15.6%) were reported having complication during prenatal period, delivery and puerperium period respectively. Moreover, 6.7% of the study participants delivered by caesarean section and instrumental. The postnatal care services were most frequently received from the health center. About 96.9% of the mothers and 68.1% of partner had tested for human immuno deficiency virus (HIV). Of them, seven mothers and five partners were infected for HIV. Nearly three-forth of the study participants received counseling services regarding postnatal care at their home by health extension workers (Table 3).

**Table 3:** Maternal health services characteristics in rural Tigray region, Northern Ethiopia, August 2017

Variables	Frequency (*n*)	Percentage (%)
Birth interval (in months) (*n* = 905)^a^		
< 24	172	15.5
24–36	565	50.9
> 36	168	15.1
ANC visit		
Yes	1018	91.8
No	91	8.2
Number of ANC visit		
No ANC visit	91	8.2
1	36	3.2
2–3	435	39.2
4^+^	547	49.3
Complication during ANC		
Yes	157	14.2
No	952	85.8
Place of delivery		
Health institution	945	85.2
Home	164	14.8
Mode of delivery (*n* = 945)		
Normal	882	79.5
Instrumental delivery (forcep,vacuums)	43	3.9
Cesarean Section (C/S)	20	1.8
Complication during delivery		
Yes	193	17.4
No	916	82.6
Where PNC received (*n* = 497)		
Health center	291	58.5
Hospital	34	6.8
Health post	172	34.7
Number of PNC visit (*n* = 497)		
1–2	417	83.9
> =3	80	16.1
Complication during postnatal period		
Yes	150	13.5
No	959	86.5
Counseled for PNC by health extension worker		
Yes	802	72.3
No	307	27.7

^a^(Among those who have previous child)

### Postpartum contraceptive use

Table 4 highlights the key descriptive statistics of current contraceptive practice, revealing that four hundred twenty five (38.3%; 95%CI: 33.8–43.5%) were currently found using modern contraception among the postpartum women. Depot medroxy progesterone acetate contraceptive and implants were the most prevalent methods of contraception use. All of the study participants received their contraception from government health facility.

**Table 4:** Descriptive statistics of current contraceptive practice of the study participants in rural Tigray region, Northern Ethiopia, August 2017

Variables	Frequency (*n*)	Percentage (%)
Current use of contraceptive		
Yes	425	38.3
No	684	61.7
Types of contraceptive use		
Injection	200	47.1
Implants (Implanon, Sinoplant and Jadelle)	125	29.4
Pills	60	14.1
IUD	40	9.4
Reason for utilization		
Spacing	390	91.7
Limiting	35	8.3
Sources of contraceptive		
Health center	225	52.9
Health post	140	32.9
Hospital	50	11.8
Private clinic	10	2.4
Intention to use contraceptive in the future		
Spacing	450	90
Limiting	50	10

### Predictors of contraceptive use

As clearly depicted on the multilevel logistic regression analysis Table 5, husband/partner education level, wealth index, postnatal care follow up, high community-level antenatal care utilization and distance from household to the health facility was independently and statistically associated with postpartum contraception use.

**Table 5:** Predictors of postpartum contraceptive use in rural Tigray region, northern Ethiopia, 2017: a multi level analysis

Determinants	Model 1	Model 2 AOR [95% CI]	Model 3 AOR [95% CI]	Model 4 AOR [95% CI]
Fixed effect				
Maternal educational-level				
Not educated	–	1	–	1
Primary-level	–	3.0 (2.1–3.0)	–	0.7 (0.6–2.1)
Secondary-level	–	2.3 (2.1–5.3)*	–	0.5 (0.4–3.2)
Diploma and above	–	3.5 (1.9–6.0)*	–	0.4 (0.3–1.3)
Partner educational-level				
Not educated	–	1	–	1
Primary-level	–	1.3 (1.2–3.2)*	–	0.9 (0.8–2.3)
Secondary-level	–	2.2 (1.5–4.4)*	–	2.3 (1.8–3.5)**
Diploma-level	–	1.4 (1.3–3.5)**	–	1.2 (1.1–2.6)***
Wealth index				
Lowest	–	1	–	1
Second	–	2. 5(1.3–4.6)*	–	0.5 (0.4–1.3)
Middle	–	2.1 (1.9–5.1)*	–	0.8 (0.7–2.4)
Fourth	–	1.3 (1.1–2.4)**	–	1.2 (1.1–3.2)***
Fifth	–	1.8 (1.5–3.2)**	–	1.5 (1.3–2.5)***
PNC	–			
Yes	–	2.3 (1.3–3.0)**	–	2.0 (1.9–4.3)***
No	–	1	–	1
Yes	–	2.7 (1.8–3.9)*	–	0.4 (0.3–2.1)
No	–	1	–	1
History of complication during delivery				
Yes	–	1.8 (1.5–2.7)*	–	0.3 (0.2–1.3)
No	–	1	–	1
Model household				
Yes	–	1.4 (1.2–3.0)*	–	0.6 (0.4–1.7)
No	–	1	–	1
Community of 4^+^ ANC use				
High	–	–	3.4 (2.4–6.1)**	2.1 (1.9–4.2)**
Low	–	–	1	1
Distance to nearest health facility				
Facility in the village	–	–	4.3 (3.9–5.4)**	3.0 (2.7–4.6)**
1 to 5 km	–	–	2.7 (1.8–5.0)*	0.6 (0.3–1.4)
> 5 km	–	–	1	1
Main sources of income				
Farming	–	–	1	1
Mixed	–	–	1.3 (1.2–3.9)*	0.4 (0.3–1.7)
Districts of residence				
Tanquae-Abrgelle	–	–	1	1
Tahtay -MayChew	–	–	0.7 (0.6–2.9)	0.5 (0.4–2.1)
LaeLay -Maychew	–	–	2.5 (2.1–3.0)*	0.7 (0.6–1.9)
Rural Adwa	–	–	3.0 (2.3–4.8)*	0.4 (0.3–2.3)
Random effects				
PCV	1	60.3	65.7	71.4
VPC	70	55.8	50.9	49.6
Community variance (SE) (Level 2)	8.07 (3.01)***	3.71 (1.14)***	3.30 (1.04)***	3.13 (0.91)***
AIC (model fit statistics)	1500	1450	1200	750

Model 1 is the null model, contained no exposure variables

Model 2 adjusted for individual-level variables

Model 3 adjusted for community-level variables

Model 4 adjusted for both individual and community-level variables

***(*p* < 0.001) **(*p* < 0.01), *p < 0.05), 1: reference category

### Individual-level effects

Women who had postnatal care visit were two times (AOR = 2.0; 95% CI: 1.9–4.3) more likely to adopt contraception in the extended postpartum period (EPP) than those who had no follow up services. The odds of contraceptive utilization were higher for postpartum women with higher socio economic status. Wealthier women who belonged to the fourth (AOR = 1.2; 95% CI: 1.1–3.2) and fifth quintile (AOR = 1.5; 95%CI: 1.3–2.5) were more likely to use postpartum modern contraception compared to women in the first, second and third quintile. Partners who attended secondary school were 2.3 times more likely to use contraception than those with no formal and primary educational-level (AOR = 2.3; 95% CI: 1.8–3.5) and those with diploma and above educational-level were 1.2 times more likely to use contraception during postpartum period (AOR = 1.2; 95% CI:1.1–2.6) (Table 5).

### Community-level effects

High community-level antenatal care utilization was 2.1 times more likely to use contraception (AOR = 2.1; 95% CI: 1.9–4.2). Women who were found proximal to the health facility (< 1 km) had higher odds to utilize contraception compared to health facility with distance more than 1 km far found from the community household (AOR = 3.0; 95% CI:2.7–4.6) (Table 5).

### Random effects

Results in Table 5, model 1 revealed that there are a variations in the probability of using postpartum contraception across communities and the variation were found significant (τ = 8.07, *p* = 0.001). The variance partition coefficient (VPC) showed that the intra-community correlation coefficient was estimated at 70%, which is variance that was explained by community-level variable. Model 2 showed the results of the effects of the individual-level variables. The intra-community correlation was 55.8% indicating that the clustering of the outcome variable (modern contraceptive use) across communities was as a result of the composition of the communities by individual-level characteristics. Model 3 the variation in use of postpartum contraception across communities remained significant. The model three shows that 50.9% of variation in the uptake of contraceptive use among the community is due to higher-level variables. The variance at the community-level in the model 4 remained significant. The intra-community correlation decreased to 49.6% indicating that the inclusion of community-level variables was important for obtaining a better explanatory model. In the model 4, the proportional change in community variance (PCV) (71.4%) of the variance in the contraceptive use across communities was due to the combination effects of lower and higher-level variables.

### Model fitness statistics

Akakie Information Criterion (AIC) was used to estimate the goodness of the fit of the adjusted final model in comparison to the preceding models (individual and community-level model adjustments). Therefore, as indicated in Table 5, the values of AIC showed subsequent decreasing from model 1 to model 4. This implies that each model represents a significant improvement over the previous model and it points out the goodness of the fit of the final model built in the multilevel analysis [34, 35].

### Multi-collnearity and interaction

Multi-collinearity was performed using the means variance inflation factor (VIF = 1.94). This means absence of any multi-collinearity between the predictor variables in the models. The presence of interaction among predictor variables was checked and there was no significant interaction between the individual and community-level variables.

## Discussion

Postpartum family planning services is one of the recommended public health intervention aimed at reducing maternal and child morbidity and mortalities [15, 36]. Therefore, determining the level and associated factors of contraceptive use among postpartum women has the potential to contribute in achieving the Ethiopian Health Sector Transformation Plan (EHSTP) [17] and to the Sustainable Development Goals (SDGs) [1] on maternal and infant survival.

The overall prevalence of the current contraception method use in the extended postpartum period was 38.3% [95% CI: 33.8–43.5%]; injectable (47.1%) and Implants (29.4%) were the most frequently use methods. In addition, pills (14.1%) and Intra-uterine contraceptive device (IUD) (9.4%) were the other least frequently used methods by the users.

This finding was higher than that of studies done among postpartum women in North West Ethiopia (10.3%) [23], Uganda (28%) [30] and India (14%) [37]. This finding might be justified by the fact that due to the efforts done by the Ethiopian government health policy to strengthen for maternal and child health morbidity and mortality reduction strategies like: the different community and institutional-based reproductive health services and health education being given by health workers, introducing women development army and community health insurance [17] might have a positive effect for the increasing use of contraceptive.

Partner/husband-level of education was positively associated with postpartum modern contraceptive. Educational status of husbands promotes the use of contraceptives. This finding parallels to studies done in North West Ethiopia [23], Jimma town [38] and India [39]. This might be explained by the fact that higher educational-level of husbands/partner plays a key role in promoting discussion, communication and support for their partner/wife for contraceptives use.

Postnatal care service use was found as a significant determinant of contraceptive uptake. A similar finding is observed in studies conducted in North Ethiopia [22], North West Ethiopia [23], Kenya and Zambia [26], India [39] and Mexico [40]. The possible explanation is that those women who attended postnatal care visit higher odds to get information toward postpartum contraception. Moreover, postnatal visit may give the opportunity for contraceptive counseling and adoption in the postpartum period.

This study indicated a direct relationship between women’s wealth status and the adoption of postpartum contraceptive. This is because rich women can provide opportunity for better information and knowledge on postpartum contraceptive methods and better access to services. Moreover, rich women are more likely to be engaged in business association and other employment activities, and thus may be more likely to want to limit their fertility. This finding is goes in line with the reports from Zambia [26], Mozambique [41] and Nigeria [42]. This study highlights the need to empower women through economic opportunities enable them to make voluntarily informed choices.

High community-level antenatal care services use were found to be significantly associated with the use of modern contraception in the extended postpartum period. The possible explanation is the study participants who have high antenatal care use in the community are more likely to get information towards family planning utilization. This relationship is consistent with the findings reported by other studies [21, 26, 40, 43, 44].

Proximity of women to health facility was the other important community-level variable which is affecting contraceptive use. This study showed strong evidence that mother who lived near the health facility are more likely to use modern contraceptive method compared to those who lived far from the health institution [45]. Higher physical accessibility may increase postpartum contraceptive uptake. This may have an implication to policy change, design important justification and implementation of appropriate interventions.

The result showed that educational status was not statistically associated with postpartum contraceptive use to what would be expected. This finding might be influenced by certain contexts. Traditional factors may affect for postpartum women and the contribution of education to contraceptive use could be low [46].

The model 4 which combined both individual and community-level variables had a best fit than model 2 and model 3. This indicated that lower and higher-level variables should be considered when studying predictors of contraption utilization among postpartum women.

### Limitations of the study

The main objective of the study was to assess the effect of individual and community-level variables on postpartum contraceptive use using a multilevel analysis. This study has some limitations that should be noted. It did not assess the whole array of determinants of contraceptive utilization; specifically, those variables related to reproductive health quality services and cultural related factors. However, we held a strong view that the inclusion of these variables wouldn’t change the main findings and subsequent conclusion as our analysis captured the most important services use measuring tools for the rural population in the study area.

### Policy implications

At the district-level, it would be helpful to look at the variations in the use of modern contraceptive during extended postpartum period and critically analyze the predictors for the differences. The differences in postpartum contraceptive uptake associated with wealth quintile, antenatal care services use, postnatal care follow up and distance from household to health facility.

## Conclusions

The status of contraceptive use is found to be low (i.e.38.3%) in rural areas of Tigray region. It was found that lower and higher-level variables showed a marked determinant on postpartum contraception use (Table 5). This study suggested that in order to increase contraceptive use the government should focus on increasing postnatal care, antenatal care services use and reduction of poverty level are important avenues for intervention.

## Abbreviations

AIC: Akaike Information Criterion
ANC: Antenatal Care
AOR: Adjusted Odds Ratio
CI: Confidence Interval
CS: Cesarean Section
DE: Design Effect
EHSTP: Ethiopian Health Sector Transformation Plan
EPP: Extended Postpartum Period
FDRE: Federal Democratic Republic of Ethiopia
HEW: Health Extension Worker
IRB: Institutional Review Board
MMR: Maternal Mortality Ratio
PCV: Proportional Change in Community Variance
PNC: Postnatal Care
PPFP: Postpartum Family Planning
SD: Standard Deviation
SDGs: Sustainable Development Goals
TRHB: Tigray Regional Health Bureau
VIF: Variance Inflation Factor
VPC: Variance Partition Coefficient
WHO: World Health Organization
